# Foot health and quality of life among adults in Riyadh, Saudi Arabia: a cross-sectional study

**DOI:** 10.1186/s13018-023-03677-w

**Published:** 2023-03-11

**Authors:** Abdulaziz Almaawi, Hashim Alqarni, Ahmed K. Thallaj, Mohammed Alhuqbani, Zyad Aldosari, Omar Aldosari, Naif Alsaber

**Affiliations:** 1 Orthopedic Surgery Department, College of Medicine, King Saud University, Riyadh, Saudi Arabia; 2 Anesthesia Department, College of Medicine, King Saud University, Riyadh, Saudi Arabia; 3 College of Medicine, King Saud University, Riyadh, Saudi Arabia

**Keywords:** Foot, Foot health, Quality of life

## Abstract

**Background:**

Foot conditions are frequent among the Saudi population. However, little is known regarding the effects of foot health on quality of life among the general Saudi population. This study aimed to assess foot health status, general health, and quality of life among the population of Riyadh using the Foot Health Status Questionnaire (FHSQ).

**Methods:**

In this cross-sectional study, out of the total number of participants approached, using a preset questionnaire, by trained medical students to participate in this study, 398 met the inclusion criteria. The questionnaire started with an informed consent followed by a set of questions regarding the sociodemographic and past medical characteristics of the participants. Foot health and overall health were assessed using a FHSQ.

**Results:**

A statistically significant positive correlation was observed between all the FHSQ domains, except for footwear. The strongest correlation was observed between foot pain and foot function, foot pain and general foot health, and foot function and general foot health. A statistically significant positive correlation was observed between general foot health and general health, vitality, social function. Our results also showed that foot pain, general foot health, vitality, and social function scores were significantly lower in women as compared to men.

**Conclusion:**

Significant positive correlation was observed between poor foot health and declining quality of life; thus, it is crucial to increase society’s awareness of the importance of medical foot care and continuous follow-up and consequences if left unrecognized and untreated. This is a major domain that can improve the well-being and quality of life of a population.

## Background

The feet are the most distinguishable enablers of bipedal locomotion. Bipedalism is a consequence of biological selection that allows greater freedom of upper limb motility, with the unintended sequelae of exerting more mechanical pressure on the lower body parts. The prevalence of degenerative joint diseases (DJDs) in weight-bearing joints suggests that human locomotion renders humans more susceptible to degenerative processes, a fact that is supplemented by the lower prevalence of DJDs in quadrupedal primates [1]. Taken together, it seems that humans are more inherently predisposed to an accelerated deterioration of locomotion, and given the incontestable importance of mobility on the quality of life (QoL), a study on foot health practices is therefore deemed warranted [2, 3].

To the best of our knowledge, no study is available regarding the effects of foot health on QoL among the general Saudi population residing in Riyadh; further, there is also a need to establish a baseline epidemiological basis for future preventive health policies. In addition, several studies are available showing the association between the disabling effects of chronic foot conditions, particularly diabetic neuropathy, on the QoL of patients with diabetes [4].

This study utilized the Foot Health Status Questionnaire (FHSQ), which is a validated tool to assess foot health in relation to QoL [5, 6]. The tool assesses the following four subscales: foot pain, foot function, footwear, and general foot health, with higher scores reflecting a more optimal status of foot health. Establishing the epidemiological background of the effect of health on the QoL is a prerequisite for the development of effective prevention policies and lays the foundation for the generation of evidence-based recommendations toward the improvement of foot health, and ultimately, health-related QoL. Our study aligns with the strategic objectives of transforming healthcare as part of Saudi Arabia’s Vision 2030 National Transformation Program aimed at improving preventive healthcare in the region [7].

## Materials and methods

### Study setting and population

This cross-sectional study was conducted between March 2022 and July 2022 to examine the adult population of Riyadh, Saudi Arabia. The participants were the citizens of Riyadh city and were approached at two locations: King Khaled University Hospital (KKUH) and a shopping mall in Riyadh. The participants were adults aged ≥ 16 years who were able to fully read and understand the questionnaire and write the appropriate response. Any participant who was under the set age or was suffering from cognitive impairment or difficulty in adequately understanding the questions was excluded from the study.

### Sample size calculation

The sample size was calculated based on the 2017 estimated population size of Riyadh Province of 8,216,284 people [8] with a 5% margin of error and 95% confidence level. Due to the limited studies on the proportion of good overall foot health status, especially in the region, the estimated proportion was set at 50% to maximize the number of enrolled participants. The final sample size included 385 participants. More than 500 participants were approached to participate in the study to compensate for refusals and incomplete responses by some participants.

### Procedure

Four hundred and ten participants agreed to participate in the study and were approached by trained medical students using a preset questionnaire designed to measure foot health status; of these, 398 met the inclusion criteria. The medical students remained with the participants throughout the answering process to ensure a full understanding of the participants for every question written. The questionnaire started with informed consent, followed by a set of questions regarding foot health and sociodemographic and past medical characteristics of the participants. Foot health and overall health were assessed using the FHSQ. The FHSQ is a self-administered questionnaire that evaluates health-related QoL [6]. It is a validated tool intended specifically for the foot, and includes three sections. The first section included 13 items related to four foot health-related subcategories: foot function, foot pain, footwear, and general foot health (Table 1). The second section contained items related to the four subscales of overall health. The third section collected sociodemographic information. The questionnaire was available in English and was translated into Arabic by two translators. The first translator had a background in instrument construction, medical language, and clinical orthopedics. The second translator had no medical background or previous experience with the instrument construct. Both versions were reviewed by specialists in the field, and following consensus, the final version was adapted. Participants were provided with a suitable language based on their preferences. To ensure participants’ anonymity, no names or IDs were identified.

**Table 1:** Domains of foot health assessed by the Foot Health Status Questionnaire

Domain	Item	Theoretical construct	Meaning of lowest score (0)	Meaning of highest score (100)
Foot pain	4	Type, severity, and duration. Evaluation of foot pain in terms of type of pain, severity, and duration	Extreme pain in the feet and significant if acute in nature	Free from pain, no discomfort
Foot function	4	Evaluation of the feet in terms of impact on physical functions	Severely limited for the performance of numerous physical activities due to their feet, such as walking, working and moving about	Patients are able to carry out all physical activities desired, such as walking, working and climbing stairs
General foot health	2	Self-perception of the feet (assessment of body image with respect to feet)	Perception of poor condition and status of the feet	Perception of excellent condition and status of the feet
Footwear	3	Lifestyle relating to footwear and feet	Great limitations to find suitable footwear	No problem obtaining suitable footwear. No limitations with respect to footwear

### FHSQ outcome measurement

This is a validated [6, 9, 10] self-administered tool on health-related QoL intended specifically for the foot. It consists of three sections that assess foot-specific and general health using version 1.03 of the FHSQ [6]. The first section consisted of 13 questions regarding four foot health-related domains: foot pain (four questions), foot function (four questions), footwear (three questions), and general foot health (two questions) (Table 1). This section has shown a high degree of content, criterion, and construct validity (Cronbach’s *α* = 0.89–0.95), as well as high retest reliability (interclass correlation coefficient = 0.74–0.92) [9]. It has been shown to be the most appropriate measurement of foot health-related QoL for a population with foot pain or pathological foot conditions and is frequently used in research and clinical settings [10–13]. The second section was measuring the health-related quality of life which was represented by: general health, physical activity, social capacity, and vigor, which were largely adapted from the Medical Outcomes Study 36-item Short-Form Health Survey [14]. Each domain was computed using statistical software and given a score ranging from 0 to 100. A score of 0 indicated a poor condition, while a score of 100 indicated the best possible condition.

### Sociodemographic and descriptive data

The sociodemographic section included age, gender, smoking status, general physical health, health insurance status, and completion of an educational certificate since leaving high school. Participants were also asked about the total number of illnesses that they were being treated for to provide a measure of comorbidity.

### Ethical consideration

This study was approved (No. E22-6679) by the institutional review board of King Saud University Medical City, Riyadh, Saudi Arabia. Participants were informed of the aims and objectives of the study, and informed consent was obtained from all participants after reassuring that their confidentiality was maintained by the exclusion of any identifying data from the questionnaire. All participants were informed of their right to withdraw from the study at any time.

### Statistical analysis

Statistical analyses were performed using R version 3.6.3. Categorical variables are summarized as counts and percentages. Continuous variables are summarized as medians ± interquartile ranges. A confirmatory analysis was performed to validate the underlying structure of the items related to foot health. Loadings > 0.5 were deemed acceptable [15]. Linear regression was used to assess the factors associated with each of the eight dimensions of the FHSQ. Cronbach’s alpha was used to assess the reliability of the foot health questionnaire [16]. Hypothesis testing was performed at the 5% significance level.

## Results

The study sample consisted of 398 participants. Of these, 56.3% were males, and 43.7% were females. After leaving school, more than three-quarters of the respondents obtained an educational degree (79.9%). Regarding medication use, 80.3% of the respondents were medically free and 21.1% reported receiving at least one medication for a chronic medical condition. One-quarter (27.1%) of the respondents reported smoking and more than one-third (36.9%) reported exercising regularly. Less than half of the respondents had private health insurance (43%) (Table 2).

**Table 2:** Descriptive statistics for the study sample

	[ALL]
	*N* = 398
Age	28.3 (9.33)
*Gender*	
Male	224 (56.3%)
Female	174 (43.7%)
*Medications*	
No meds	314 (80.3%)
One condition	59 (15.1%)
Two conditions	18 (4.60%)
*Smoking*	
No	290 (72.9%)
Yes	108 (27.1%)
*Regular exercise*	
No	251 (63.1%)
Yes	147 (36.9%)
*Private health insurance*	
No	227 (57.0%)
Yes	171 (43.0%)
*Educational qualification since leaving school*	
No	80 (20.1%)
Yes	318 (79.9%)

Data was summarized using counts and percentages

**Fig. 1:**
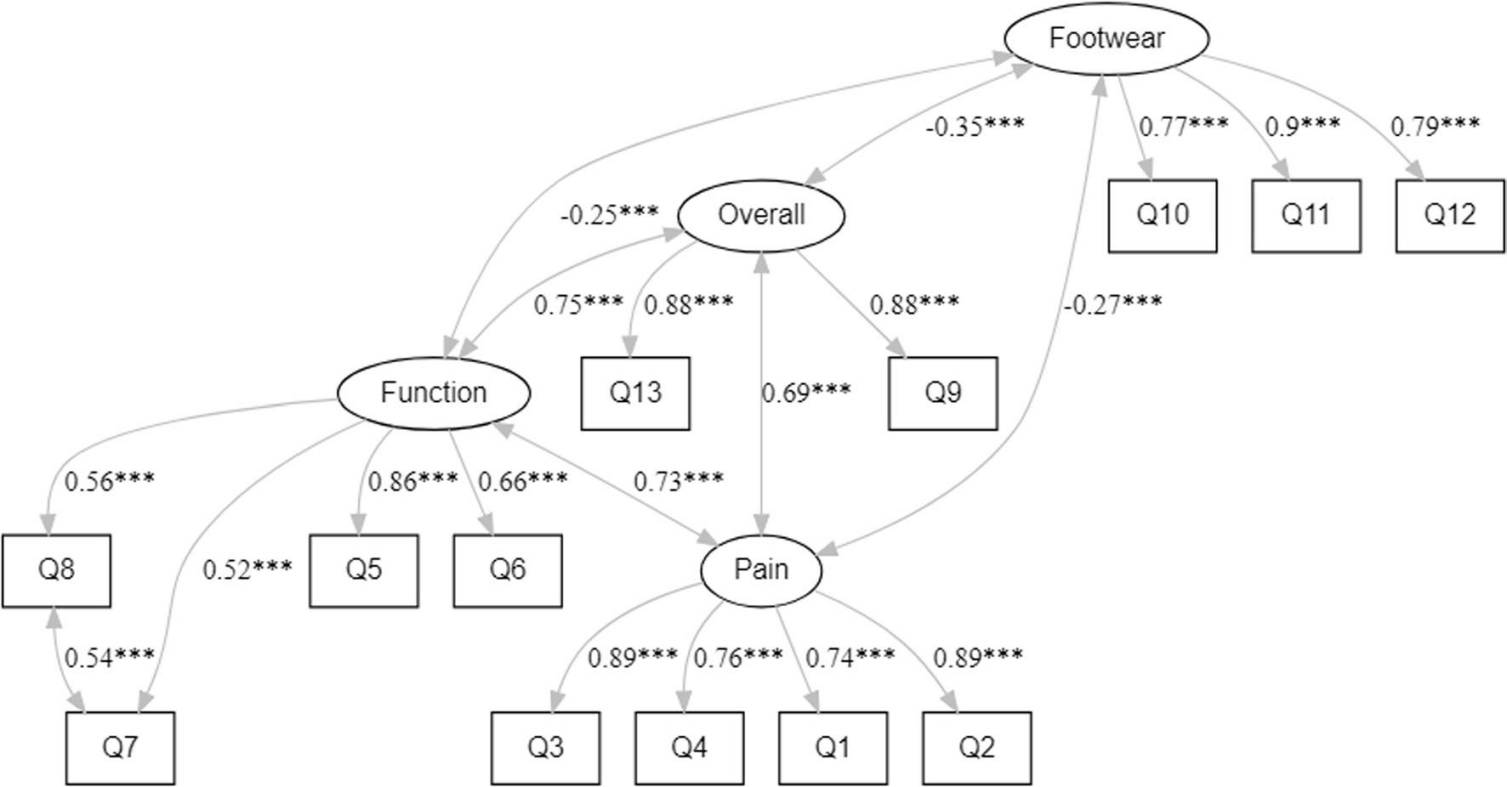
Confirmatory factor analysis results

The results showed that all loadings were > 0.7 (Fig. 1), which was considered excellent. The only exception was the functional domain, although the loadings were > 0.5, which was still above the minimally acceptable threshold of 0.5. A statistically significant positive correlation was observed between the four dimensions of the FHSQ foot health. The correlation was the lowest between footwear and the other three dimensions (Table 3).

**Table 3:** Coefficients of correlation between FHSQ questionnaire scales

	Physical function	Footwear	Social Function	Vitality	General health	Foot Function	General Foot health	Foot pain
Foot pain	0.134**	0.251***	0.277***	0.282***	0.289***	0.580***	0.625***	–
General Foot health	0.156**	0.303***	0.368***	0.381***	0.359***	0.572***	–	–
Foot Function	0.226***	0.175***	0.257***	0.248***	0.360***	–	–	–
General health	0.303***	0.063	0.265***	0.358***	–	–	–	–
Vitality	0.156**	0.095	0.401***	–	–	–	–	–
Social Function	0.139**	0.246***	–	–	–	–	–	–
Footwear	0.038	–	–	–	–	–	–	–
Physical function	–	–	–	–	–	–	–	–

*P < 0.05; **P < 0.01; ***P < 0.001

The reliability of the four dimensions was assessed using Cronbach’s alpha (α) and was > 0.7 for all four scales, which was above the minimum acceptable threshold (Table 4).

**Table 4:** Item internal consistency and factor loadings

Foot pain domain	Pain	Function	Footwear	Overall health
What level of foot pain have you had during the past week?	0.735			
How often have you had foot pain?	0.886			
How often did your feet ache?	0.893			
How often did you get sharp pains in your feet?	0.759			
*Foot function domain*				
Have your feet caused you to have difficulties in your work or activities?		0.855		
Were you limited in the kind of work you could do because of your feet?		0.657		
How much does your foot health limit you in walking?		0.519		
How much does your foot health limit you from climbing stairs?		0.564		
*Footwear domain*				
It is hard to find shoes that do not hurt my feet			0.771	
I have difficulty finding shoes that fit my feet			0.896	
I am limited in the number of shoes I can wear			0.788	
*General foot health domain*				
How would you rate your overall foot health?				0.878
In general, what condition would you say your feet are in?				0.877
Cronbach’s alpha	0.89	0.8	0.86	0.87

A statistically significant positive correlation was observed between all the FHSQ domains, except for footwear. No association was observed between footwear and the three overall health dimensions of vitality, general health, and physical function. The highest correlation was observed between foot pain and foot function (*r* = 0.58, *P* < 0.001), foot pain and general foot health (*r* = 0.625, *P* < 0.001), and foot function and general foot health (*r* = 0.572, *P* < 0.001). A statistically significant positive correlation was observed between foot health and general health (*r* = 0.359, *P* < 0.001) (Table 3).

A statistically significant decreasing linear trend was observed in foot pain, foot function, general foot health, and footwear scores with increasing number of medications (Table 5). Smoking was associated with a lower score in the foot pain domain (*B* = −6.12, *P* = 0.023), but not in other domains. Neither health insurance nor regular exercise was significantly associated with scores for any domain. Better education was associated with a higher score in the foot function domain (*B* = 8.75, *P* = 0.001), but not in the other domains.

**Table 5:** Factors associated with foot health

Predictors	Foot pain		Foot function		General foot health		Footwear	
	*B* (95% CI)	*P*	*B* (95% CI)	*P*	*B* (95% CI)	*P*	*B* (95% CI)	*P*
*Gender*								
Male	Reference		Reference		Reference		Reference	
Female	− 8.48 (− 13.21 to − 3.75)	< 0.001	− 3.12 (− 7.34 to 1.11)	0.148	− 6.96 (− 11.42 to − 2.51)	0.002	− 5.80 (− 12.29 to 0.69)	0.079
*Medications*								
No meds	Reference		Reference		Reference		Reference	
One condition	− 13.19 (− 19.36 to − 7.01)	< 0.001	− 6.78 (− 12.30 to − 1.27)	0.016	− 7.99 (− 13.81 to − 2.18)	0.007	− 6.36 (− 14.82 to 2.11)	0.141
Two conditions	− 17.30 (− 28.03 to − 6.57)	0.002	− 11.33 (− 20.92 to − 1.74)	0.021	− 20.15 (− 30.25 to − 10.05)	< 0.001	− 16.29 (− 31.01 to − 1.58)	0.030
*Smoking*								
No	Reference		Reference		Reference		Reference	
Yes	− 6.12 (− 11.38 to − 0.86)	0.023	− 3.03 (− 7.73 to 1.67)	0.206	− 2.61 (− 7.57 to 2.34)	0.300	− 2.92 (− 10.13 to 4.30)	0.427
*Regular exercise*								
No	Reference		Reference		Reference		Reference	
Yes	− 2.49 (− 7.21 to 2.23)	0.300	− 1.23 (− 5.45 to 2.98)	0.565	1.16 (− 3.28 to 5.61)	0.607	0.30 (− 6.17 to 6.77)	0.927
*Health insurance*								
No	Reference		Reference		Reference		Reference	
Yes	0.60 (− 3.94 to 5.15)	0.794	− 0.25 (− 4.31 to 3.82)	0.905	− 2.85 (− 7.12 to 1.43)	0.192	− 6.01 (− 12.24 to 0.22)	0.059
*Education*								
No	Reference		Reference		Reference		Reference	
Yes	5.16 (− 0.48 to 10.79)	0.073	8.75 (3.72 to 13.79)	0.001	4.64 (− 0.66 to 9.94)	0.086	− 0.25 (− 7.98 to 7.48)	0.949

High scores indicate a better health status in all four domains

Only sex showed a statistically significant association with vitality (*B* = − 4.82, *P* = 0.011), with females showing lower scores compared to males. Gender also showed a statistically significant association with social functioning (*B* = − 6.52, *P* = 0.011). The median foot pain score was significantly lower in females than males with a median score of 78.12 and 71.88 for males and females, respectively (*P* = 0.009). The results for the general foot health, foot function, footwear, and vigor domains were not dissimilar, as all showed a significantly higher score for males than for females. Our results were equivocal with respect to the physical and social activity domains, lacking a statistically significant difference between the two groups (Table 6). The use of medications was not associated with physical functioning but showed a statistically significant association with social functioning. A statistically significant decrease in the score was observed with an increase in the use of medications from no medications to one (*B* =  − 12.12, *P* < 0.001) and two (*B* =  − 18.46, *P* = 0.002) medications. Neither smoking nor health insurance was associated with the four general health domains. Regular exercise was associated with higher scores on the physical function domain (*B* = 9.75, *P* = 0.003). Education was associated with a higher score in the general health (*B* = 5.3, *P* = 0.038) and physical function (*B* = 8.69, *P* = 0.027) domains (Table 7).

**Table 6:** Comparisons of the FHSQ scores between males and females in the study sample

FHSQ domains	Total group Median ± IR (Range) *n* = 398	Male Median ± IR (Range) *n* = 224	Female Median ± IR (Range) *n* = 174	*P* value male versus female*
Foot pain	75.00 ± 28.13 (0–100)	78.12 ± 33.75 (6.25–100)	71.88 ± 30.63 (0–100)	0.008504
Foot function	93.75 ± 25.00 (0–100)	93.75 ± 25.00 (0–100)	87.50 ± 25.00 (12.50–100)	0.06109
Footwear	85.00 ± 27.5 (0–100)	92.50 ± 20.00 (0–100)	85.00 ± 40.00 (0–100)	0.000852
General foot health	66.67 ± 50.00 (0–100)	66.67 ± 58.32 (0–100)	59.24 ± 50.00 (0–100)	0.04657
General health	70.00 ± 30.00 (20–100)	70.00 ± 30.00 (20–100)	70.00 ± 30.00 (20–100)	0.5914
Physical activity	75.00 ± 50.00 (0–100)	77.78 ± 55.56 (0–100)	72.22 ± 44.44 (0–100)	0.4739
Social activity	75.00 ± 50.00 (0–100)	75.00 ± 37.50 (0–100)	75.00 ± 37.50 (0–100)	0.01304
Vigor	56.25 ± 50.00 (0–100)	56.25 ± 18.75 (18.75–100)	50.00 ± 18.75 (6.25–100)	0.01264

*FHSQ* Foot Health Status Questionnaire, *IR* Interquartile range

*Median ± interquartile range, range (min–max), and Mann–Whitney test were used (99% confidence interval; *P* < 0.01 is considered statically significant)

**Table 7:** Factors associated with general health

Predictors	General health		Physical function		Social function		Vitality	
	*B* (95% CI)	*P*	*B* (95% CI)	*P*	*B* (95% CI)	*P*	*B* (95% CI)	*P*
*Gender*								
Male	Reference		Reference		Reference		Reference	
Female	− 1.54 (− 5.74 to 2.65)	0.470	0.65 (− 5.79 to 7.10)	0.842	− 6.52 (− 11.56 to − 1.48)	0.011	− 4.82 (− 8.55 to − 1.10)	0.011
*Medications*								
No meds	Reference		Reference		Reference		Reference	
One condition	− 7.15 (− 12.63 to − 1.68)	0.011	0.30 (− 8.12 to 8.71)	0.945	− 12.12 (− 18.70 to − 5.55)	< 0.001	− 4.13 (− 9.00 to 0.73)	0.096
Two conditions	− 4.51 (− 14.04 to 5.01)	0.352	− 3.77 (− 18.40 to 10.86)	0.613	− 18.46 (− 29.89 to − 7.02)	0.002	− 2.85 (− 11.30 to 5.61)	0.509
*Smoking*								
No	Reference		Reference		Reference		Reference	
Yes	− 3.37 (− 8.04 to 1.29)	0.156	1.32 (− 5.86 to 8.49)	0.718	0.84 (− 4.77 to 6.44)	0.769	0.98 (− 3.17 to 5.12)	0.643
*Regular exercise*								
No	Reference		Reference		Reference		Reference	
Yes	1.79 (− 2.40 to 5.97)	0.402	9.75 (3.31 to 16.18)	0.003	1.20 (− 3.83 to 6.23)	0.639	2.94 (− 0.78 to 6.66)	0.121
*Health insurance*								
No	Reference		Reference		Reference		Reference	
Yes	0.15 (− 3.89 to 4.18)	0.943	1.11 (− 5.08 to 7.31)	0.724	− 0.11 (− 4.95 to 4.73)	0.964	0.08 (− 3.50 to 3.66)	0.963
*Education*								
No	Reference		Reference		Reference		Reference	
Yes	5.30 (0.30 to 10.30)	0.038	8.69 (1.00 to 16.37)	0.027	− 0.33 (− 6.34 to 5.67)	0.913	0.17 (− 4.27 to 4.61)	0.940

High scores indicate a better health status in all four domains

## Discussion

This study aimed to determine the relationship between QoL and foot health among adults living in Riyadh, Saudi Arabia. The evidential effect of foot health on QoL also manifests itself in the degree of physical activity performed by individuals, which would ultimately be conducive to a healthier, more physically active lifestyle. Based on different studies of the population of Riyadh, the reported prevalence of different foot conditions is higher than that in other countries. For instance, it was reported that the prevalence of hallux valgus in the region was around 43% which was higher than the global prevalence (23%) [17].

This study gauges the perception of the enrolled participants as it pertains to foot health and well-being. Foot problems may plausibly affect QoL in the personal, social, and occupational domains. Our results demonstrated a correlation between higher general health score and better foot pain, foot function, and foot health domain scores indicating a greater quality of life. Furthermore, less foot pain was positively correlated with foot function, which enables greater control and autonomy over one’s lifestyles, and physical activity [18, 19]. Moreover, the results showed that foot pain, general foot health, vitality score, and social function score were significantly lower in females compared to males. Further, foot function and footwear were lower but not significant. Our results were consistent with previous reports, where lower scores were observed among females, particularly in the foot health-related domains, which was most significant in the general foot health and foot pain domains [20–23]. The aforementioned findings are suggestive of lower QoL as it pertains to foot health among females in our cohort, which may be explained by the higher prevalence of foot conditions among Saudi female population [17, 24]. Moreover, it is well documented that females are more keen about their appearance and foot health. A meta-analysis found that the majority of female participants were more active in watching over their feet, shoes, and slippers and demanded more professional assistance with foot care than that of male participants [25]. Further studies should scrupulously analyze the lifestyle and innate differences that contribute to these dissimilarities in order to formulate targeted therapeutic interventions or lifestyle changes for such disparities.

Previous studies have explored the impact of chronic medical conditions and different foot health and function parameters on general health, physical and social function, and QoL. Many of these studies have discussed chronic medical conditions such as diabetes [20, 26, 27], obesity [20], osteoarthritis [27], hemophilia [28], and even physiological changes in pregnancy [29] and menopause [30] or various foot diseases [2, 12, 20, 31], presenting with pain or foot dysfunction that ultimately and negatively impacts a person’s QoL. This could be avoided if those causes or risk factors are addressed early and managed, which will help to improve general health, QoL, and ultimately autonomy.

This study had few limitations. First, the scarcity of research addressing foot pain and deformities in the region made it difficult to formulate rigorous methodologies to minimize the confounding effects of foot conditions on the outcome variables. Second, the FHSQ is based on the subjective feelings of participants without any objective clinical correlation to solidify the presence of recognizable foot pathologies. Finally, the study settings were not suitable to access all age groups, especially older adults. A more diverse study setting is required to access enough participants of different age groups to ascertain sex-based differences across multiple age groups and further interpretation of associated factors that could impact various foot health parameters to better understand how foot health affects QoL.

## Conclusion

Our results emphasize the need for further research on foot problems in Riyadh region of Saudi Arabia. Clear associations were shown between poor foot health and declining QoL; thus, it is crucial to increase the awareness of society on the importance of medical foot care and continuous follow-up and the consequences of not addressing this issue. Hence, proper foot care and maintaining a better foot health represent major domains for improving the well-being and QoL of a population.

## Data Availability

The datasets used during the current study are available from the corresponding author on reasonable request.

## Abbreviations

FHSQ: Foot Health Status Questionnaire
DJDs: Degenerative joint diseases
QoL: Quality of life
